# Seroprevalence of IgG antibodies against measles in adolescents and adults from Metropolitan Lima and Callao, Peru, 2024-2025

**DOI:** 10.17843/rpmesp.2026.433.16877

**Published:** 2026-09-17

**Authors:** Natalia Vargas-Herrera, Margot Vidal-Anzardo, Chandel Farroñay-García, Nestor E. Cabezudo Pillpe, Roxana Ramírez-Salcedo, Edda Salamanca-Pacheco, Myriam Yasuda-Espinoza, Víctor Fiestas-Solórzano

**Affiliations:** 1 Centro Nacional de Salud Pública, Instituto Nacional de Salud, Lima, Peru. Centro Nacional de Salud Pública Instituto Nacional de Salud Lima Peru; 2 Unidad de Intervenciones Estratégicas, Instituto Nacional de Salud, Lima, Peru. Unidad de Intervenciones Estratégicas Instituto Nacional de Salud Lima Peru; 3 Laboratorio de Referencia Nacional de Patología Clínica, Instituto Nacional de Salud, Lima, Peru. Laboratorio de Referencia Nacional de Patología Clínica Instituto Nacional de Salud Lima Peru; 4 Área de Sarampión y Rubéola, Laboratorio de Referencia Nacional de Virus Inmunoprevenibles, Instituto Nacional de Salud, Lima, Peru. Área de Sarampión y Rubéola Laboratorio de Referencia Nacional de Virus Inmunoprevenibles Instituto Nacional de Salud Lima Peru

**Keywords:** Measles, Seroprevalence, Young Adult, Immunoglobulin G, Vaccination, Peru

## Abstract

**Objectives.:**

To estimate the seroprevalence of measles IgG antibodies in people aged 15 years or older residing in Lima Metropolitan and Callao, Peru, through secondary analysis of serum samples obtained from the National Institute of Health's Non-Communicable Diseases Surveillance between 2024 and 2025.

**Materials and methods.:**

A descriptive, population-based, cross-sectional study that included 770 serum samples, in which the presence of measles IgG antibodies was determined using a commercial ELISA assay. Global seroprevalence was estimated and stratified by sex, age group, and origin.

**Results.:**

After data expansion, 53.6% of the studied population corresponded to women. The global seroprevalence was 82.1% (95% CI: 78.6%-85.1%). Adults aged 20 to 29 years showed the lowest seroprevalence (55.6%), followed by adolescents aged 15 to 19 years (72.0%). Seroprevalence progressively increased with age and exceeded 95% in people aged 50 years or older. No significant differences were observed by sex or area of origin.

**Conclusions.:**

Immunity gaps against measles are evident mainly in young adults, in a context of disease resurgence in the Americas and an ongoing outbreak in Peru. These findings highlight the value of seroprevalence studies to identify susceptible groups and guide public health interventions, such as targeted vaccination strategies, strengthening epidemiological surveillance, and information campaigns to reduce immunity gaps and contribute to maintaining measles elimination in Peru.

## INTRODUCTION

Measles is a highly transmissible infectious disease, with an estimated basic reproductive number (R0) between 12 and 18, which continues to represent a threat to global public health, despite the availability of safe and effective vaccines ^1^. During recent years, the decline in vaccination coverage in various countries has favored the resurgence of outbreaks and compromised the progress made in the elimination of the disease ^2^. The Region of the Americas officially achieved the elimination of endemic measles transmission in 2016; however, the sustained increase in cases observed since 2024 has compromised this achievement. As of epidemiological week (EW) 23 of 2026, more than 22 000 confirmed cases and 38 deaths were reported in 17 countries ^3^, with the vast majority of these cases concentrated in Mexico (n=11532), Guatemala (n=6895), and the United States (n=2097). Several of these countries, although they maintain elimination status, face a growing risk of losing it, such as Canada in November 2025 after registering more than 6000 cases ^4^. Consequently, the Region of the Americas lost its elimination status in 2025, after more than 12 months of endemic virus transmission. In response, the Pan American Health Organization (PAHO) urges countries to strengthen regional surveillance and vaccination strategies with the aim of regaining elimination and preventing new outbreaks ^5^.

In Peru, the elimination of the disease was consolidated in March 2000, when the last autochthonous case was confirmed. Since then and until 2025, reported cases were mainly imported. However, in 2026 community transmission of the virus was confirmed, with more than 90% of cases concentrated in the Puno region. Until EW 26, more than 800 cases had been confirmed, which placed Peru among the five countries with the highest disease burden in the Region of the Americas ^6^^,^^7^. Faced with this scenario, the Ministry of Health (MINSA) issued epidemiological alerts aimed at strengthening vaccination activities, epidemiological surveillance, and control measures to limit the spread of the virus to other regions of the country ^8^^,^^9^.

The regular vaccination schedule in the country includes the administration of two doses of the measles-mumps-rubella (MMR) vaccine, at 12 and 18 months of age. In the absence of serological studies, population protection against measles is estimated indirectly from the administrative vaccination coverages. According to MINSA, MMR coverages during the 2020-2025 period were lower than recommended by the World Health Organization (WHO) to maintain elimination and prevent outbreaks (95%). Although the coverage of the first dose reached 97.6% in 2024 after the national sweep campaigns carried out in 2023 and 2024, the coverage of the second dose only reached 83.2% in 2024 and 82.1% in 2025 ^10^. This situation suggests a progressive accumulation of susceptible individuals and a higher risk of transmission upon virus introduction.

The detection of IgG antibodies by the ELISA technique is widely used to assess population immunity in various infectious diseases ^11^. In epidemic outbreak scenarios, it constitutes an indirect marker of protective immunity that allows identifying immunity gaps according to age groups and other epidemiological characteristics of the population ^1^. The WHO recommends the periodic conduct of measles and rubella seroprevalence studies as a complement to surveillance systems and administrative vaccination coverages to identify population immunity gaps ^12^. However, the information concerning the immune response against measles in our country is scarce and is focused on the pediatric population. In that context, Fiestas *et al*. in 2011 reported a national seroprevalence of 91.6% in children aged 1 to 4 years ^13^.

The present study is justified by the increase in measles cases and the risk of reintroduction and dissemination of the disease in susceptible populations. In this scenario, it is a priority to have updated evidence on the level of seropositivity against measles in the population, especially in age groups where immunity gaps might exist. Therefore, the objective of the study was to estimate the seroprevalence of IgG antibodies against measles in people aged 15 years and older from Metropolitan Lima and Callao, using serum samples previously obtained within the framework of laboratory surveillance of non-communicable diseases. The results will allow identifying population groups with a lower proportion of detectable antibodies and providing evidence to guide decision-makers and public health authorities in strengthening, focusing, and prioritizing vaccination strategies, contributing to closing immunity gaps and reducing the risk of measles transmission.

**KEY MESSAGES**

Motivation for the study. To have updated and local information on the population's protection level against measles, and to identify the groups with the highest risk of falling ill and spreading the disease, given its resurgence in Peru and the Americas.

Main findings. The immune response against measles in the studied population was below the recommended level to prevent transmission and the occurrence of outbreaks, especially in adults under 50 years of age.

Public health implications. These findings highlight the importance of strengthening vaccination and epidemiological and laboratory surveillance, prioritizing the most susceptible groups.

## MATERIALS AND METHODS

### Study design

A descriptive cross-sectional population-based study, based on the secondary analysis of serum samples obtained within the framework of the Laboratory Surveillance of Non-Communicable Diseases, implemented by the Subdirectorate of Research and Laboratories of Non-Communicable Diseases (SUDENT) of the National Institute of Health (INS), in coordination with the National Institute of Statistics and Informatics (INEI). This laboratory surveillance used a subsample of individuals aged 15 years or older from the probabilistic sample of the 2024 Demographic and Family Health Survey (ENDES) in Metropolitan Lima and Callao. Participant recruitment was carried out among individuals interviewed in the 2024 ENDES during the period from May to September of that year.

### Study population

The study population consisted of individuals aged 15 years and older, from Lima Metropolitan and Callao, from whom whole blood samples were obtained between July 15, 2024, and April 27, 2025, within the framework of the Laboratory Surveillance of Non-communicable Diseases (NCD) carried out by the INS through home visits. This surveillance was conducted in a subsample of participants from ENDES 2024, which uses a probabilistic, complex, and multi-stage sampling design.

As part of the INEI program, initially 4700 households were selected by ENDES 2024, identifying 4033 eligible individuals aged 15 years or older, of whom 3423 were interviewed during 2024. The sub-sample selection period was coordinated with the ENDES sampling team, including only participants surveyed between May and September 2024 from the ENDES annual sample. During this period, 1230 eligible individuals were identified, of whom 1212 agreed to be contacted by INS, ultimately contacting 1115 individuals. Of these, 781 individuals agreed to participate and underwent blood sample collection. Finally, of the 781 serum samples obtained, 770 met the inclusion criteria of the present study and were considered for the analysis of IgG antibody seroprevalence against measles (Figure 1 and 2).

**Figure 1:**
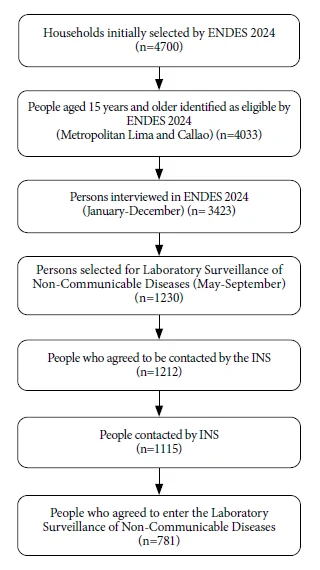
Flowchart of selection of the subsample of the Laboratory Surveillance of Non-Communicable Diseases from ENDES 2024.

ENDES: Demographic and Family Health Survey, INS: National Institute of Health.

**Figure 2:**
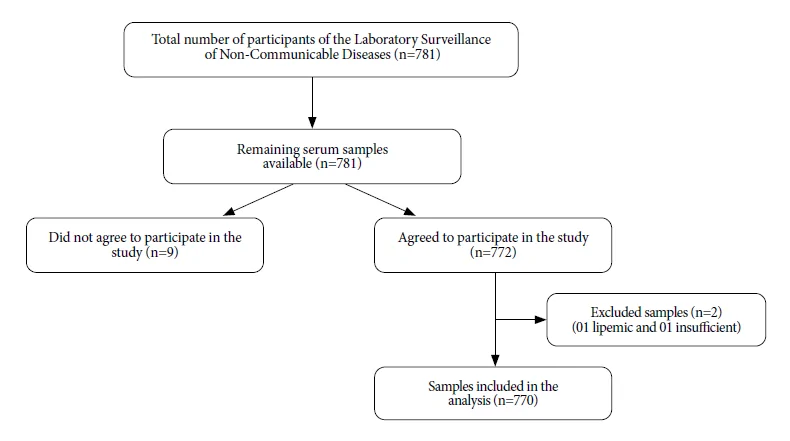
Flowchart of sample selection for seroprevalence analysis of IgG antibodies against measles.

The most frequent reason for non-participation in the laboratory surveillance of NCDs by the INS was the refusal to obtain biological samples. This subsample maintained the probabilistic properties and the capacity for population inference due to the use of specific weighting factors according to sex and age.

Whole blood samples were centrifuged for serum collection and subsequently stored under established conditions for their preservation. All available serum samples were used from participants who, through informed consent, authorized the storage and use of the remaining samples in future research on public health diseases of interest.

### Criteria for inclusion and exclusion

Serum samples were included with sufficient remnant volume for measles IgG antibody determination, with available information on age, sex, and origin, and adequately preserved according to the conditions established by the biobank or storage system.

Samples with hemolysis, lipemia, or deterioration that could compromise the validity of the assay were excluded, as well as those from participants who did not authorize the secondary use of their samples.

### Sample size

The present study used a subsample from ENDES 2024, which was initially estimated for NCD Laboratory Surveillance, for this reason, it was calculated based on the lowest prevalence of the conditions of interest for said surveillance (expected prevalence of diabetes 7%), a design effect of 1.15, a maximum coefficient of variation (CV) of 14%, and a non-response rate of 30%, therefore, for said surveillance only an effective sample size of 780 was needed, with only 770 samples available and eligible for analysis. In order to evaluate if the sample size was adequate for the assessment of IgG antibody seroprevalence against measles, a sample size calculation was performed using the Epi Info^TM^7 program considering an approximate reference population of 8.4 million people aged 15 years or older from Metropolitan Lima and Callao (according to INEI demographic projections), an expected seroprevalence of 80%, a 95% confidence level, a 4% margin of error, and a design effect of 2. With these assumptions, a minimum required sample size of 768 participants was obtained, the 770 eligible and available samples being sufficient for the present analysis.

### Variables

The outcome variable was the result of IgG antibody serology against measles, classified as positive, negative, or indeterminate according to the cut-off points established by the manufacturer.

Stratification variables were sex, age group, categorized into 15 to 19 years, 20 to 29, 30 to 39, 40 to 49, 50 to 59, and ≥ 60 years, and place of origin classified according to the Integrated Health Network Directorates (DIRIS) of Metropolitan Lima and the Regional Health Directorate (DIRESA) of Callao.

### Procedures

As part of the NCD Laboratory Surveillance conducted by the INS, the whole blood samples were centrifuged at 3,500 rpm for 10 minutes to separate the serum from the red blood cells, which was aliquoted into specific coded cryovials for the sample and kept frozen at -20 °C in the National Reference Laboratory of Clinical Pathology of the National Center for Public Health (CNSP) of the INS from the date the sample was obtained. Before processing, the macroscopic appearance of the sera was evaluated, excluding those highly hemolyzed or lipemic. Subsequently, the samples were thawed and sent under refrigeration to the National Reference Laboratory of Immunopreventable Viruses to be processed using the commercial ELISA technique, VIRCELL ^14^^)^ brand, for the detection of IgG antibodies against the measles virus, following the manufacturer's instructions. According to the test's technical sheet, it presents a diagnostic sensitivity of 97% and a specificity of 94%. In each analytical run, internal quality controls were incorporated, including a negative control, a cut-off control, and a positive control, to ensure test validation and guarantee the reliability of the obtained results.

The processing was performed in the Measles and Rubella Area of the National Reference Laboratory for Immunopreventable Viruses of the CNSP of the INS, by personnel competent in serological tests, complying with biosafety level 2 regulations, the constant use of personal protective equipment (PPE), and the management of biocontaminated solid waste according to current regulations (Technical Health Standard N° 144-MINSA/2018/DIGESA). All samples with initially indeterminate results were reprocessed only once using the same ELISA kit, following the manufacturer's instructions. Samples that remained indeterminate after the process were classified and reported as such in the final analysis, as well as in the corresponding denominator for the results report.

### Statistical analysis

The results were recorded in a Microsoft Excel database, using an identification code assigned to each sample. Statistical processing and analysis were performed using Stata version 14 software (College Station, TX: StataCorp LLC. 2019). Previously, quality control of the database was performed in order to verify the consistency of the information and reduce possible typing or classification errors.

The analyses incorporated the complex sample design of ENDES 2024 using the specific expansion factors of the Laboratory Surveillance (FVL) provided by INEI. The details of the formulas and procedures applied for the expansion are shown in the supplementary material.

For the specification of the sample design in Stata, the variables sample weight, stratum, and cluster were used using the commands *svyset* and *svy*. The configuration of the sample design was performed using the cluster as the primary sampling unit, the sample weight as the weighting factor, and the stratum as the stratification variable, with the aim of obtaining representative estimates of the target population and considering the structure of the complex sample design in the calculation of estimates, their standard errors, and confidence intervals. Considering the 30% non-response rate, the ENDES-INEI team developed calibrated sample weights for the subsample, using official population projections for Metropolitan Lima and Callao for 2024, by sex and age groups. This allowed reducing biases derived from differential non-response and voluntary participation in biomedical procedures, improving the representativeness of the subsample with respect to the reference population.

Only estimates with CV≤15% were presented, in order to ensure an acceptable level of statistical precision and population inference, following the methodological criterion used by ENDES 2024, which considers that estimates with CV<5% have very good precision, from 5% to <10% have good precision, from 10% to <15% acceptable precision, and ≥15% is only referential ^(^^15^. No estimates without an acceptable level of statistical precision were found.

For the descriptive analysis, categorical variables were presented using unweighted absolute frequencies, expanded frequencies, and weighted proportions, with their respective 95% confidence intervals. Age was categorized into age groups, therefore, no normality assessment was performed. Results were presented using tables, bar charts, and line charts.

The seroprevalence of IgG antibodies against measles was estimated globally and stratified by sex, age group, and place of origin (DIRIS Lima Centro, Lima Sur, Lima Este, Lima Norte, and DIRESA Callao). The age variable was categorized into age groups.

For bivariate analysis, the corrected chi-square test for complex sample designs was used to evaluate differences in the seroprevalence of IgG antibodies against measles by sex, age group, and origin. Since statistically significant differences were only observed by age group, multiple pairwise comparisons were performed among the six age groups using the *pwcompare* command in Stata with Bonferroni correction. Fifteen pairwise comparisons were performed, and statistical significance was considered when the adjusted p-value was less than 0.05.

### Ethical Considerations

The research protocol for this study received approval from the Institutional Ethics Committee of the National Institute of Health, and was assigned approval code OI-005-26. Only serum samples were analyzed that had signed informed consent for use in future research on relevant public health diseases.

## RESULTS

770 biological samples were analyzed from participants aged 15 years and older from Metropolitan Lima and Callao, which, after applying sampling weights, represented an estimated population of 9,195,834 people. According to weighted estimates, 53.6% corresponded to women (4,928,298 people) and 46.4% to men (4,267,536 people); in unweighted terms, the sample included 479 women and 291 men.

44.0% of the represented population corresponded to the 15-to-39-year-old group (4,050,446 individuals). Regarding the area of origin, 10.4% corresponded to DIRESA Callao (expanded n = 957,804), 14.5% to DIRIS Lima Centro (1,336,797), 20.7% to DIRIS Lima Sur (1,899,676), 25.2% to DIRIS Lima Norte (2,316,697), and 29.2% to DIRIS Lima Este (2,684,859) (table 1).

**Table 1:** Sociodemographic characteristics.

Characteristic		Sample	Population^a^	Weighted %
Sex				
	Men	291	4 267 536	46.4
	Women	479	4 928 298	53.6
Age group				
	From 15 to 19 years	72	803 694	8.7
	From 20 to 29 years	137	1 422 980	15.5
	30 to 39 years	237	1 823 772	19.8
	From 40 to 49 years	134	1 923 406	20.9
	50 to 59 years	80	1 200 193	13.1
	60 or more	110	2 021 789	22.0
DIRIS/DIRESA				
	Callao	235	957 804	10.4
	Lima Centro	83	1 336 797	14.5
	Lima Este	186	2 684 859	29.2
	Lima Norte	149	2 316 697	25.2
	Lima Sur	117	1 899 676	20.7
	Total	770	9 195 834	100.0

DIRIS: Integrated Health Network Directorates, DIRESA: Regional Health Directorate.

a Population estimate obtained through sample weights.

The overall seroprevalence of IgG antibodies against measles was 82.1% (95% CI: 78.6%-85.1%), with a CV of 1.9%, indicating adequate precision of the estimate (table 2).

**Table 2:** Seroprevalence of IgG antibodies against measles in people aged 15 years or older from Lima Metropolitan and Callao, 2024-2025, according to sociodemographic characteristics.

Variable		n	Population^a^	Seroprevalence %	95%CI	CV	Value of p^b^
Total		770	9 195 834	82.1	78.6-85.1	1.9	—
Sex							0.983
	Women	479	4 928 298	82.0	78.1-85.4	2.3	
	Men	291	4 267 536	82.1	76.6-86.5	3.1	
Age group							<0.001
	15-19 years	72	803 694	72.0	58.1-82.7	8.7	
	20-29 years	137	1 422 980	55.6	45.9-64.7	8.6	
	30-39 years	237	1 823 772	76.7	70.6-81.9	3.7	
	40-49 years	134	1 923 406	85.2	76.8-90.9	4.1	
	50-59 years	80	1 200 193	95.1	83.9-98.6	3.3	
	≥60 years	110	2 021 789	98.8	91.6-99.8	1.2	
DIRIS/DIRESA							0.768
	Callao	235	957 804	81.4	75.4-86.2	3.3	
	Lima Centro	83	1 336 797	84.7	75.6-90.8	4.5	
	Lima Este	186	2 684 859	80.8	73.9-86.2	3.9	
	Lima Norte	149	2 316 697	84.2	77.7-89.1	3.4	
	Lima Sur	117	1 899 676	79.7	69.5-87.1	5.6	

CV: Coefficient of variation, DIRIS: Integrated Health Networks Directorates, DIRESA: Regional Health Directorate.

a Population estimate obtained using the sample weights.

b Corrected chi-square test for complex design.

The seroprevalence of IgG antibodies against measles was similar by sex. In women it was 82.0% (95% CI: 78.1%-85.4%; CV: 2.3%), and in men 82.1% (95% CI: 76.6%-86.5%; CV: 3.1%), with no statistically significant differences evidenced between both groups (table 2).

The seroprevalence of IgG antibodies against measles in the evaluated population showed a heterogeneous pattern according to age group. The lowest seroprevalences were observed in the 20 to 29 years age group (55.6%; 95% CI: 45.9%-64.7%; CV: 8.6%) and the 15 to 19 years age group (72.0%; 95% CI: 58.1%-82.7%; CV: 8.7%). From 30 years of age, a progressive increase in seroprevalence was evidenced, reaching 76.7% in the 30 to 39 years age group, 85.2% in the 40 to 49 years age group, 95.1% in those 50 to 59 years of age, and 98.8% in people aged 60 years and over. All estimates presented coefficients of variation lower than 10% (table 2, supplementary material).

The seroprevalence of IgG antibodies against measles was also similar among the evaluated DIRIS and DIRESA. The highest estimates were recorded in Lima Centro (84.7%; 95% CI: 75.6%-90.8%; CV: 4.5%), and Lima Norte (84.2%; 95% CI: 77.7%-89.1%; CV: 3.4%), followed by Callao (81.4%; 95% CI: 75.4%-86.2%; CV: 3.3%) and Lima Este (80.8%; 95% CI: 73.9%-86.2%; CV: 3.9%). The lowest seroprevalence was observed in Lima Sur, with 79.7% (95% CI: 69.5%-87.1%; CV: 5.6%). Overall, the estimates ranged from 79.7% and 84.7% without significant differences (table 2, supplementary material).

## DISCUSSION

During the last few years, measles vaccination coverages have notably decreased worldwide, compromising collective immunity against this disease and favoring the reappearance of outbreaks on all continents, including the region of the Americas ^16^.

In this context, the overall seroprevalence of IgG antibodies against measles observed in our study was below the population immunity threshold recommended by the WHO (95%) to interrupt sustained virus transmission and maintain the disease elimination status ^17^. This finding evidences the persistence of immunity gaps in the studied population, since approximately one in five individuals did not present detectable IgG antibodies against measles. While the absence of detectable IgG does not necessarily imply absolute susceptibility due to the role of cellular immunity and neutralizing antibodies not evaluated in this study, the results suggest the existence of population groups potentially vulnerable to infection and to the spread of the virus in case of exposure ^18^.

The results obtained are comparable with those recently reported in other countries. In Mexico, a global seroprevalence of 78.3% ^19^ was estimated, while in Costa Rica seropositivity reached 74.8% ^20^. Similarly, studies conducted in Italy and Spain reported seroprevalences of 91.2% and 92.2%, respectively ^(^^21^^,^^22^. Collectively, these findings demonstrate that immunity gaps against measles persist even in countries that have achieved or maintained disease elimination, highlighting the need to strengthen vaccination and population immunity monitoring strategies.

When analyzing the seroprevalence of IgG antibodies against measles by age groups, a marked heterogeneity was observed. Adolescents and adults under 40 years of age showed the lowest levels of seropositivity, with the 20-29 year age group recording the lowest proportion of detectable antibodies. From this group onwards, seroprevalence progressively increased with age, exceeding 95% in people aged 50 years or older. These findings are of great relevance for public health, since young adults usually show greater mobility and social interaction due to work, academic, migratory, and tourist activities, which could favor the introduction and spread of the virus. Likewise, this distribution is consistent with the latest reports from the measles situational room of the Centro Nacional de Epidemiología, Prevención y Control de Enfermedades, (CDC Perú), which show a higher proportion of confirmed cases in young adults ^6^. A similar pattern has been described in seroprevalence studies conducted in Costa Rica, México, and Italy ^19^.

Various factors could explain the lower seropositivity observed in adolescents and young adults. These cohorts were born during a period of epidemiological transition characterized by a progressive decrease in measles virus circulation as a consequence of the introduction and expansion of vaccination ^20^. Consequently, they likely had less exposure to the circulating virus and, therefore, fewer opportunities to experience natural immune boosting phenomena. In Peru, the measles vaccine was incorporated into the Expanded Program on Immunization (EPI) in the 1970s ^23^^,^^24^; as a result, a significant proportion of these cohorts would have developed their immunity primarily through vaccination. Unlike natural infection, vaccine-induced immunity can be associated with a progressive decrease in detectable antibody levels over time in the absence of subsequent exposures to the virus, and a negative result does not necessarily indicate absence of protection ^25^. Additionally, other factors could have contributed, such as incomplete vaccination schedules, considering that the systematic introduction of two doses of MMR vaccine was only massively introduced in Peru in 2006 ^26^, as well as primary vaccine response failures.

In contrast, the high seropositivity observed in older adults could be explained by the immunity acquired after natural infection, which is characterized by more robust and durable humoral responses that can persist throughout life ^27^. This is probably because these cohorts were born during periods of high viral circulation, prior to the introduction of measles vaccination, so they had a higher probability of exposure and infection during childhood ^28^. Furthermore, as previously mentioned, the measles vaccine only became available during the 1970s, so a large proportion of people currently over 50 years old grew up in a context of endemic viral circulation. Similar findings have been reported in studies conducted in Thailand, Mexico, and Costa Rica, where older adults showed the highest proportions of seropositivity compared to other age groups ^29^. When analyzed by sex, no statistically significant differences were found in the seroprevalence of male and female participants, nor when evaluating by participants' place of origin.

The results of this study evidence immunity gaps against measles in young people and adults from Metropolitan Lima and Callao. Although the absence of detectable IgG antibodies does not necessarily imply absolute susceptibility, the findings suggest the existence of cohorts with protection levels lower than those recommended to prevent sustained virus transmission. In the current context of community transmission in Peru, this situation could increase the risk of outbreaks occurring and spreading.

Therefore, these findings highlight the need to prioritize vaccination strategies in groups with larger immunity gaps, particularly young adults, while maintaining high coverage in the child population. Furthermore, they demonstrate that the recovery and sustainability of measles elimination require not only high and homogeneous vaccination coverage, but also robust epidemiological surveillance systems and timely response to outbreaks.

Thus, the reemergence of measles reveals that, to recover the elimination status of this disease at the regional level, it is essential, but not sufficient, to maintain high and homogeneous vaccination coverages. These coverages must be sustained over time and complemented with robust epidemiological and laboratory surveillance, effective alert-response systems for outbreaks and emergencies, the promotion of global disease elimination, and the maintenance of follow-up campaigns ^30^. These strategies, adopted by PAHO in 1996, allowed for the interruption of endemic virus circulation in a large part of the Americas and achieved the lowest number of cases registered until then in the region ^(^^31^.

In this regard, seroprevalence studies are fundamental as they provide information on population immunity, as well as an estimate of the burden of disease. Furthermore, they contribute to the evaluation of the effectiveness of immunization programs by acting as indirect indicators of vaccination coverage, facilitating the identification of immunity gaps in specific groups and guiding targeted vaccination interventions in this population ^(^^20^^,^^32^. In that sense, these studies should be replicated in other regions of Peru to obtain a more precise estimate of seroprevalence at the national level.

The present study has some limitations that are described below. First, the analysis corresponds to a subsample of ENDES 2024 participants who voluntarily agreed to participate in the INS Laboratory Surveillance of Non-Communicable Diseases. Although the original ENDES design was probabilistic and the analyses incorporated specific expansion factors adjusted for non-response and calibrated with population projections by sex and age, a selection bias or residual non-response cannot be completely ruled out. Second, participant recruitment took place between May and September 2024 and not throughout the entire ENDES 2024 data collection period. While the selected clusters were distributed geographically and throughout the considered period homogeneously, the existence of differences between the included participants and those interviewed during other months of the year cannot be completely excluded. Third, the estimates obtained are representative of the population aged 15 years or older from Lima Metropolitan and Callao considered together, and not necessarily from each DIRIS or DIRESA Callao independently. Consequently, disaggregated estimates for these jurisdictions should be interpreted with caution as subgroup analyses. Similarly, the results should not be directly extrapolated to the rest of Peru due to regional differences in demographic structure, vaccination coverage, socioeconomic conditions, population mobility, and historical measles circulation patterns. Fourth, the cross-sectional design of the study reflects population immunity during the second half of 2024 and the first four months of 2025, which might not reflect the current reality. Fifth, immunity was assessed by detecting IgG antibodies using the ELISA method, a technique widely used in seroepidemiological studies, including COVID-19 ^33^. Neutralizing antibodies or cellular immunity were not included, so the results might underestimate the actual immunological protection of some individuals. Finally, information regarding the vaccination history against measles of the studied population was not available, so it could not be established with certainty whether the observed seropositivity was due to a response from natural infection or vaccination.

However, one of the main strengths of this study is that it constitutes the most recent source of information on measles seroprevalence in Peru, which is of great importance in the current epidemiological context. Additionally, it mainly includes young and adult populations, groups rarely studied in measles seroprevalence research, both nationally and internationally. Finally, the stratified analysis by age groups allowed identifying immunity gaps in specific cohorts, generating useful evidence to guide and prioritize focused vaccination strategies.

In conclusion, the results of this study indicate that young adults constitute an important group of susceptible individuals by presenting the lowest levels of immunity against measles, particularly individuals aged 20 to 29 years. These findings highlight the need to prioritize prevention strategies aimed at this population, which include focused vaccination interventions and the strengthening of epidemiological surveillance through active case finding nationwide, and information campaigns that promote vaccine confidence. The timely closure of these immunity gaps will be fundamental to control the current outbreak in our country, stop endemic measles transmission and preserve the sustained elimination status achieved so far.
