# Supplementary material for: Seroprevalence of IgG antibodies against measles in adolescents and adults from Metropolitan Lima and Callao, Peru, 2024-2025
Source: Rev Peru Med Exp Salud Publica. 2026 Sep 17;43(3):301–10. doi: 10.17843/rpmesp.2026.433.16877 (PMC13718565; doi:10.17843/rpmesp.2026.433.16877)
Supplement: Supplementary file 1 [file rpmesp-43-03-16877-s001-es.docx]

**MATERIAL SUPLEMENTARIO**

**Anexo 1**

**Anexo 2**

**Procedimientos aplicados para la expansión muestral**

El FVL se construyó a partir del inverso de la probabilidad de selección de las unidades de muestreo en las diferentes etapas, incorporando los ajustes correspondientes por la no respuesta de la ENDES 2024 y de la Vigilancia Laboratorial, mediante la formula:

Donde P1 corresponde a la probabilidad de selección del conglomerado (unidad primaria de muestreo), P2 a la probabilidad de selección de la vivienda (unidad secundaria de muestreo), P3 a la probabilidad de selección de una persona de 15 años o más dentro del hogar, TNR_E a la tasa de no respuesta de la ENDES y TNR_VL a la tasa de no respuesta de la Vigilancia Laboratorial.

Posteriormente, el factor de expansión fue calibrado mediante postestratificación simple, utilizando como totales de control las proyecciones de población del INEI al 30 de junio de 2024, según las combinaciones de sexo y grupo de edad. El factor de expansión calibrado para cada participante se calculó mediante la siguiente formula:

donde FVLᵢ corresponde al factor de expansión de la Vigilancia Laboratorial antes de la calibración; FVLᵢ^calibrada^, al factor de expansión calibrado correspondiente a la persona i; PobProyec^INEI^_(s,g)_, a la población proyectada por el INEI para la combinación de sexo (s) y grupo de edad (g) al 30 de junio de 2024; y PobExp^Vigilancia^ _(s,g)_, a la población estimada mediante la expansión de la muestra de la Vigilancia Laboratorial para la misma combinación de sexo y grupo de edad. De esta manera, la suma de los factores de expansión calibrados reproduce los totales poblacionales de referencia establecidos por el INEI para cada dominio de sexo y grupo de edad. Las proyecciones poblacionales utilizadas corresponden a las proyecciones oficiales de población del INEI al 30 de junio de 2024, disponibles en la colección institucional Estimaciones y proyecciones de población nacional, departamental, provincial y distrital del INEI.
