# Supplementary material for: Seroprevalence of IgG antibodies against measles in adolescents and adults from Metropolitan Lima and Callao, Peru, 2024-2025
Source: Rev Peru Med Exp Salud Publica. 2026 Sep 17;43(3):301–10. doi: 10.17843/rpmesp.2026.433.16877 (PMC13718565; doi:10.17843/rpmesp.2026.433.16877)
Supplement: Translation

# Translation: Seroprevalencia de anticuerpos IgG contra el sarampión en adolescentes y adultos de Lima Metropolitana y Callao, Perú, 2024-2025

**Authors:** Natalia Vargas-Herrera, Margot Vidal-Anzardo, Chandel Farroñay-García, Nestor E. Cabezudo Pillpe, Roxana Ramírez-Salcedo, Edda Salamanca-Pacheco, Myriam Yasuda-Espinoza, Víctor Fiestas-Solórzano

**Affiliations:** 1 Centro Nacional de Salud Pública, Instituto Nacional de Salud, Lima, Perú.; 2 Unidad de Intervenciones Estratégicas, Instituto Nacional de Salud, Lima, Perú.; 3 Laboratorio de Referencia Nacional de Patología Clínica, Instituto Nacional de Salud, Lima, Perú.; 4 Área de Sarampión y Rubéola, Laboratorio de Referencia Nacional de Virus Inmunoprevenibles, Instituto Nacional de Salud, Lima, Perú.

**Keywords:** Sarampión, Seroprevalencia, Anticuerpos, Vacunación, Inmunidad Colectiva, Perú

## Abstract

**Objetivos.:**

Estimar la seroprevalencia de anticuerpos IgG contra el sarampión en personas de 15 años o más residentes en Lima Metropolitana y Callao, Perú, mediante el análisis secundario de muestras de suero obtenidas en la Vigilancia de Enfermedades no Transmisibles del Instituto Nacional de Salud entre el 2024 y 2025.

**Materiales y métodos.:**

Estudio descriptivo transversal de base poblacional que incluyó 770 muestras de suero, en las que se determinó la presencia de anticuerpos IgG contra el sarampión mediante un ensayo ELISA comercial. Se estimó la seroprevalencia global y estratificada por sexo, grupo etario y procedencia.

**Resultados.:**

Tras la expansión de los datos, el 53,6% de la población estudiada correspondió a mujeres. La seroprevalencia global fue de 82,1% (IC95%: 78,6%-85,1%). Los adultos de 20 a 29 años presentaron la menor seroprevalencia (55,6%), seguido por los adolescentes de 15 a 19 años (72,0%). La seroprevalencia aumentó progresivamente con la edad y supero el 95% en personas de 50 años o más. No se observaron diferencias significativas según sexo ni área de procedencia.

**Conclusiones.:**

Se evidencian brechas de inmunidad contra el sarampión principalmente en adultos jóvenes, en un contexto de resurgimiento de la enfermedad en las Américas y de un brote actual en Perú. Estos hallazgos resaltan el valor de los estudios de seroprevalencia para identificar grupos susceptibles y orientar intervenciones de salud pública, como estrategias de vacunación focalizadas, el fortalecimiento de la vigilancia epidemiológica y campañas de información para reducir las brechas de inmunidad y contribuir a mantener la eliminación del sarampión en el Perú.

## INTRODUCCIÓN

El sarampión es una enfermedad infecciosa altamente transmisible, con un número reproductivo básico (R0) estimado entre 12 y 18, que continúa representando una amenaza para la salud pública mundial, a pesar de la disponibilidad de vacunas seguras y efectivas ^1^. Durante los últimos años, la disminución de las coberturas de vacunación en diversos países ha favorecido el resurgimiento de brotes y comprometido los avances alcanzados en la eliminación de la enfermedad ^2^. La Región de las Américas alcanzó oficialmente la eliminación de la transmisión endémica del sarampión en 2016; sin embargo, el incremento sostenido de casos observado desde 2024 ha comprometido dicho logro. Hasta la semana epidemiológica (SE) 23 del 2026 se notificaron más de 22 000 casos confirmados y 38 defunciones en 17 países ^3^, concentrándose la gran mayoría de estos casos en México (n=11 532), Guatemala (n=6895) y Estados Unidos (n=2097). Varios de estos países, aunque mantienen el estatus de eliminación, enfrentan un riesgo creciente de perderlo, como Canadá en noviembre del 2025 después de registrar más de 6000 casos ^4^. Como consecuencia, la Región de las Américas, perdió el estatus de eliminación en el año 2025, después de más de 12 meses de transmisión endémica del virus. En respuesta, la Organización Panamericana de la Salud (OPS) insta a los países a reforzar las estrategias regionales de vigilancia y vacunación con el objetivo de recuperar la eliminación y prevenir nuevos brotes ^5^.

En el Perú, la eliminación de la enfermedad se consolidó en marzo del 2000, cuando se confirmó el último caso autóctono. Desde entonces y hasta el 2025, los casos notificados fueron principalmente importados. Sin embargo, en 2026 se confirmó la transmisión comunitaria del virus, con más del 90% de los casos concentrados en la región Puno. Hasta la SE 26, se habían confirmado más de 800 casos, lo que ubico al Perú entre los cinco países con mayor carga de enfermedad en la Región de las Américas ^6^^,^^7^. Frente a este escenario, el Ministerio de Salud (MINSA) emitió alertas epidemiológicas orientadas a fortalecer las actividades de vacunación, la vigilancia epidemiológica y las medidas de control para limitar la propagación del virus hacia otras regiones del país ^8^^,^^9^.

El esquema regular de vacunación en el país contempla la administración de dos dosis de la vacuna sarampión-rubéola-paperas (SRP), a los 12 y 18 meses de edad. En ausencia de estudios serológicos, la protección poblacional frente al sarampión se estima indirectamente a partir de las coberturas administrativas de vacunación. Según el MINSA, las coberturas de SRP durante el período 2020-2025 fueron inferiores al recomendado por la Organización Mundial de la Salud (OMS) para mantener la eliminación y evitar brotes (95%). Aunque la cobertura de la primera dosis alcanzó el 97,6% en 2024 tras las campañas de barrido nacional realizadas en 2023 y 2024, la cobertura de la segunda dosis sólo alcanzó 83,2% en 2024 y 82,1% en 2025 ^10^. Esta situación sugiere una acumulación progresiva de personas susceptibles y un mayor riesgo de transmisión ante la introducción del virus.

La detección de anticuerpos IgG por la técnica ELISA es ampliamente utilizada para evaluar la inmunidad poblacional en diversas enfermedades infecciosas ^11^. En escenarios de brotes epidémicos constituye un marcador indirecto de inmunidad protectora que permite identificar brechas de inmunidad según grupos etarios y otras características epidemiológicas de la población ^1^. La OMS recomienda la realización periódica de estudios de seroprevalencia de sarampión y rubéola como complemento a los sistemas de vigilancia y a las coberturas administrativas de vacunación para identificar brechas de inmunidad poblacional ^12^. No obstante, la información concerniente a la respuesta inmune contra el sarampión en nuestro país es escasa y está enfocada en población pediátrica. En ese contexto, Fiestas *et al*. en el 2011 reportaron una seroprevalencia nacional de 91,6% en niños de 1 a 4 años de edad ^13^.

El presente estudio se justifica por el incremento de casos de sarampión y el riesgo de reintroducción y diseminación de la enfermedad en poblaciones susceptibles. En este escenario, resulta prioritario contar con evidencia actualizada sobre el nivel de seropositividad frente al sarampión en la población, especialmente en grupos etarios en los que podrían existir brechas de inmunidad. Por ello, el objetivo del estudio fue estimar la seroprevalencia de anticuerpos IgG contra el sarampión en personas de 15 años a más de Lima Metropolitana y Callao, utilizando muestras de suero obtenidas previamente en el marco de la vigilancia laboratorial de enfermedades no transmisibles. Los resultados permitirán identificar grupos poblacionales con menor proporción de anticuerpos detectables y brindar evidencia para orientar a los decisores y autoridades de salud pública en el fortalecimiento, focalización y priorización de las estrategias de vacunación, contribuyendo al cierre de brechas de inmunidad y a la reducción del riesgo de transmisión del sarampión.

**MENSAJES CLAVE**

Motivación para realizar el estudio. Contar con información actualizada y local sobre el nivel de protección de la población frente al sarampión, e identificar los grupos con mayor riesgo de enfermar y diseminar la enfermedad, ante su resurgimiento en el Perú y las Américas.

Principales hallazgos. La respuesta inmune contra el sarampión en la población estudiada estuvo por debajo del nivel recomendado para prevenir la transmisión y la aparición de brotes, especialmente en los adultos menores de 50 años.

Implicancias en salud pública. Estos hallazgos resaltan la importancia de fortalecer la vacunación y vigilancia epidemiológica y laboratorial, priorizando a los grupos más susceptibles.

## MATERIALES Y MÉTODOS

### Diseño de estudio

Estudio descriptivo transversal de base poblacional, basado en el análisis secundario de muestras de suero obtenidas en el marco de la Vigilancia Laboratorial de Enfermedades No Transmisibles, implementada por la Subdirección de Investigación y Laboratorios de Enfermedades No Transmisibles (SUDENT) del Instituto Nacional de Salud (INS), en coordinación con el Instituto Nacional de Estadística e Informática (INEI). Esta vigilancia laboratorial utilizó una submuestra de personas de 15 años o más procedente de la muestra probabilística de la Encuesta Demográfica y de Salud Familiar (ENDES) 2024 en Lima Metropolitana y Callao. La captación de participantes se realizó entre las personas entrevistadas en la ENDES 2024 durante el periodo comprendido entre mayo a setiembre de ese año.

### Población de estudio

La población de estudio estuvo conformada por personas de 15 años a más, procedentes de Lima Metropolitana y Callao, de quienes se obtuvo muestras de sangre total entre el 15 de julio de 2024 y el 27 de abril de 2025, en el marco de la Vigilancia Laboratorial de Enfermedades No Transmisibles (ENT) que efectúa el INS mediante visitas domiciliarias. Esta vigilancia se realizó en una submuestra de participantes de la ENDES 2024, la cual emplea un diseño muestral probabilístico, complejo y multietápico.

Como parte de la programación del INEI, inicialmente se seleccionaron 4700 viviendas por la ENDES 2024, identificándose 4033 personas elegibles de 15 años o más, de las cuales 3423 fueron entrevistadas durante 2024. El periodo de selección de la submuestra fue coordinado con el equipo de muestreo de la ENDES, incluyendo únicamente a los participantes encuestados entre mayo y septiembre del 2024 de la muestra anual de la ENDES. En este periodo se identificaron 1230 personas elegibles, de las cuales 1212 aceptaron ser contactadas por el INS logrando finalmente el contactar a 1115 personas. De éstas, 781 personas aceptaron participar y se les realizó la toma de muestra sanguínea. Finalmente, de las 781 muestras de suero obtenidas, 770 cumplieron con los criterios de inclusión del presente estudio y fueron consideradas para el análisis de seroprevalencia de anticuerpos IgG contra el sarampión (figura 1 y 2).

**Figura 1:** Flujograma de selección de la submuestra de la Vigilancia Laboratorial de Enfermedades No Transmisibles a partir de la ENDES 2024.

ENDES: Encuesta Demográfica y de Salud Familiar, INS: Instituto Nacional de Salud.

**Figura 2:** Flujograma de selección de muestras para análisis de seroprevalencia de anticuerpos IgG contra el sarampión.

El motivo más frecuente de no participación en la Vigilancia laboratorial de ENT del INS fue la negativa a la obtención de muestras biológicas. Esta submuestra mantuvo las propiedades probabilísticas y la capacidad de inferencia poblacional debido al uso de factores de ponderación específicos según sexo y edad.

Las muestras de sangre total fueron centrifugadas para la obtención de suero y posteriormente almacenado bajo condiciones establecidas para su conservación. Se utilizó la totalidad de muestras de suero disponibles de participantes que, mediante consentimiento informado, autorizaron el almacenamiento y uso de las muestras remanentes en futuras investigaciones sobre enfermedades de interés en salud pública.

### Criterios de inclusión y exclusión

Se incluyeron muestras de suero con volumen remanente suficiente para la determinación de anticuerpos IgG contra el sarampión, con información disponible de edad, sexo y procedencia, y conservadas adecuadamente según las condiciones establecidas por el biobanco o sistema de almacenamiento.

Se excluyeron muestras con hemolisis, lipemia o deterioro que pudieran comprometer la validez del ensayo, así como aquellas provenientes de participantes que no autorizaron el uso secundario de sus muestras.

### Tamaño de muestra

El presente estudio utilizó una submuestra de la ENDES 2024, la cual se estimó inicialmente para la Vigilancia Laboratorial de ENT, por tal motivo, fue calculada en base a la menor prevalencia de las condiciones de interés de dicha vigilancia (prevalencia esperada de diabetes 7%), un efecto de diseño de 1,15, un coeficiente de variación (CV) máximo de 14% y una tasa de no respuesta de 30%, por lo tanto, para dicha vigilancia sólo se necesitó un tamaño efectivo de muestra de 780, contando sólo con 770 muestras disponibles y elegibles para el análisis. Con el fin de evaluar si el tamaño de muestra era adecuado para la evaluación de seroprevalencia de anticuerpos IgG contra el sarampión, se realizó un cálculo de tamaño de muestra mediante el programa Epi Info^TM^7 considerando una población de referencia aproximada de 8,4 millones de personas de 15 años o más procedentes de Lima metropolitana y Callao (de acuerdo a las proyecciones demográficas del INEI), una seroprevalencia esperada de 80%, un nivel de confianza de 95%, un margen de error de 4% y un efecto de diseño de 2. Con estos supuestos se obtuvo un tamaño de muestra mínimo requerido de 768 participantes, siendo suficientes las 770 muestras elegibles y disponibles para el presente análisis.

### Variables

La variable de desenlace fue el resultado de la serología por anticuerpos IgG contra el sarampión, clasificado como positivo, negativo o indeterminado según los puntos de corte establecidos por el fabricante.

Las variables de estratificación fueron el sexo, grupo etario, categorizado en 15 a 19 años, 20 a 29, 30 a 39, 40 a 49, 50 a 59 y ≥ 60 años, y el lugar de procedencia, clasificado según las Direcciones de Redes Integradas de Salud (DIRIS) de Lima Metropolitana y la Dirección Regional de Salud (DIRESA) Callao.

### Procedimientos

Como parte de la Vigilancia Laboratorial de ENT que realiza el INS, las muestras de sangre total fueron centrifugadas a 3500 rpm durante 10 minutos para separar el suero del paquete globular, el cual fue alicuotado en crioviales codificados específicos para la muestra y conservadas en congelación a -20 °C en el Laboratorio de Referencia Nacional de Patología Clínica del Centro Nacional de Salud Pública (CNSP) del INS desde la fecha de obtención de la muestra. Antes del procesamiento se evaluó el aspecto macroscópico de los sueros, excluyendo aquellos altamente hemolizados o lipémicos. Posteriormente, las muestras fueron descongeladas y enviadas en refrigeración al Laboratorio de Referencia Nacional de Virus Inmunoprevenibles para ser procesadas mediante la técnica de ELISA comercial, marca VIRCELL ^14^^)^ para la detección de anticuerpos IgG contra el virus del sarampión, siguiendo las instrucciones del fabricante. De acuerdo con la ficha técnica de la prueba, ésta presenta una sensibilidad diagnóstica del 97% y una especificidad del 94%. En cada corrida analítica se incorporaron controles internos de calidad, incluyendo un control negativo, un control de punto de corte (cut-off) y un control positivo, para garantizar la validación de la prueba y asegurar la confiabilidad de los resultados obtenidos.

El procesamiento fue realizado en el Área de Sarampión y Rubéola del Laboratorio de Referencia Nacional de Virus inmunoprevenibles del CNSP del INS, por personal con competencias en pruebas serológicas, cumpliendo las normas de bioseguridad nivel 2, el uso en todo momento del equipo de protección personal (EPP), y el manejo de residuos sólidos biocontaminados según la normativa vigente (Norma Técnica de Salud N° 144-MINSA/2018/DIGESA). Todas las muestras con resultados inicialmente indeterminados fueron reprocesadas una sola vez utilizando el mismo kit ELISA, siguiendo las instrucciones del fabricante. Las muestras que permanecieron con resultado indeterminado tras el proceso fueron clasificadas e informadas como tal en el análisis final, así como en el denominador correspondiente para el reporte de los resultados.

### Análisis estadístico

Los resultados fueron registrados en una base de datos en Microsoft Excel, utilizando un código de identificación asignado a cada muestra. El procesamiento y análisis estadístico se realizó con el programa Stata versión 14 (College Station, TX: StataCorp LLC. 2019). Previamente, se realizó el control de calidad de la base de datos, con el fin de verificar la consistencia de la información y reducir posibles errores de digitación o clasificación.

Los análisis incorporaron el diseño muestral complejo de la ENDES 2024 mediante los factores de expansión específicos de la Vigilancia Laboratorial (FVL) proporcionados por el INEI. Los detalles de las fórmulas y procedimientos aplicados para la expansión se muestran en el material suplementario.

Para la especificación del diseño muestral en Stata se usaron las variables peso muestral, estrato y conglomerado mediante los comandos *svyset* y *svy.* La configuración del diseño de muestra se realizó utilizando el conglomerado como unidad primaria de muestreo, el peso muestral como factor de ponderación y el estrato como variable de estratificación, con el objetivo de obtener estimaciones representativas de la población objetivo y considerar la estructura del diseño muestral complejo en el cálculo de las estimaciones, sus errores estándar e intervalos de confianza. Considerando la tasa de no respuesta de 30%, el equipo de la ENDES-INEI elaboró pesos muestrales calibrados para la submuestra, utilizando proyecciones poblacionales oficiales de Lima Metropolitana y Callao para el 2024, según sexo y grupos de edad. Esto permitió reducir sesgos derivados de la no respuesta diferencial y de la participación voluntaria en los procedimientos biomédicos, mejorando la representatividad de la submuestra respecto de la población de referencia.

Se presentaron únicamente las estimaciones con CV≤15%, a fin de asegurar un nivel aceptable de precisión estadística e inferencia poblacional, siguiendo el criterio metodológico empleado por la ENDES 2024, el cual considera que las estimaciones con CV<5% tienen una muy buena precisión, de 5% a <10% tienen buena precisión, de 10% a <15% precisión aceptable y ≥15% es sólo referencial ^(^^15^. No se encontraron estimaciones sin un nivel aceptable de precisión estadística.

Para el análisis descriptivo, las variables categóricas fueron presentadas mediante frecuencias absolutas no ponderadas, frecuencias expandidas y proporciones ponderadas, con sus respectivos intervalos de confianza al 95%. La edad fue categorizada en grupos etarios, por lo que no se realizó evaluación de normalidad. Los resultados fueron presentados mediante tablas, gráficos de barras y gráficos de líneas.

La seroprevalencia de anticuerpos IgG contra el sarampión se estimó de manera global y estratificada según sexo, grupo etario y lugar de procedencia (DIRIS Lima Centro, Lima Sur, Lima Este, Lima Norte y DIRESA Callao). La variable edad fue categorizada en grupos etarios.

Para el análisis bivariado se utilizó la prueba de chi cuadrado corregida para diseños muestrales complejos, para evaluar diferencias en la seroprevalencia de anticuerpos IgG contra el sarampión según sexo, grupo etario y procedencia. Dado a que sólo se observaron diferencias estadísticamente significativas según grupo etario, se realizaron comparaciones múltiples por pares entre los seis grupos de edad mediante el comando *pwcompare* en Stata con corrección de Bonferroni. Se efectuaron 15 comparaciones pareadas y se consideró significancia estadística cuando el valor de p ajustado fue menor a 0,05.

### Consideraciones éticas

El protocolo de investigación de este estudio recibió aprobación por parte del Comité Institucional de ética del Instituto Nacional de Salud, y se le asignó el código de aprobación OI-005-26. Sólo se analizaron las muestras de suero que contaban con consentimiento informado firmado para uso en investigaciones futuras de enfermedades relevantes de salud pública.

## RESULTADOS

Se analizaron 770 muestras biológicas de participantes de 15 años a más de Lima Metropolitana y Callao, las cuales, tras aplicar los pesos muestrales, representaron a una población estimada de 9 195 834 personas. Según las estimaciones ponderadas, el 53,6% correspondió a mujeres (4 928 298 personas) y el 46,4% a hombres (4 267 536 personas); en términos no ponderados, la muestra incluyó 479 mujeres y 291 hombres.

El 44,0% de la población representada correspondió al grupo de 15 a 39 años (4 050 446 personas). Respecto al ámbito de procedencia, el 10,4% correspondió a la DIRESA Callao (n expandido = 957 804), el 14,5% a la DIRIS Lima Centro (1 336 797), el 20,7% a la DIRIS Lima Sur (1 899 676), el 25,2% a la DIRIS Lima Norte (2 316 697) y el 29,2% a la DIRIS Lima Este (2 684 859) (tabla 1).

**Tabla 1:** Características sociodemográficas.

Característica		Muestra	Población^a^	% ponderado
Sexo				
	Hombres	291	4 267 536	46,4
	Mujeres	479	4 928 298	53,6
Grupo etario				
	De 15 a 19 años	72	803 694	8,7
	De 20 a 29 años	137	1 422 980	15,5
	De 30 a 39 años	237	1 823 772	19,8
	De 40 a 49 años	134	1 923 406	20,9
	De 50 a 59 años	80	1 200 193	13,1
	De 60 a más	110	2 021 789	22,0
DIRIS/DIRESA				
	Callao	235	957 804	10,4
	Lima Centro	83	1 336 797	14,5
	Lima Este	186	2 684 859	29,2
	Lima Norte	149	2 316 697	25,2
	Lima Sur	117	1 899 676	20,7
	Total	770	9 195 834	100,0

DIRIS: Direcciones de Redes Integradas de Salud, DIRESA: Dirección Regional de Salud.

a Estimación poblacional obtenida mediante los pesos muestrales.

La seroprevalencia general de anticuerpos IgG contra el sarampión fue de 82,1% (IC95%: 78,6%-85,1%), con un CV de 1,9%, lo que indica una adecuada precisión de la estimación (tabla 2).

**Tabla 2:** Seroprevalencia de anticuerpos IgG contra sarampión en personas de 15 años o más de Lima Metropolitana y Callao, 2024-2025, según características sociodemográficas.

Variable		n	Población^a^	Seroprevalencia %	IC95%	CV	Valor de p^b^
Total		770	9 195 834	82,1	78,6-85,1	1,9	-
Sexo							0,983
	Mujeres	479	4 928 298	82,0	78,1-85,4	2,3	
	Hombres	291	4 267 536	82,1	76,6-86,5	3,1	
Grupo etario							<0,001
	15-19 años	72	803 694	72,0	58,1-82,7	8,7	
	20-29 años	137	1 422 980	55,6	45,9-64,7	8,6	
	30-39 años	237	1 823 772	76,7	70,6-81,9	3,7	
	40-49 años	134	1 923 406	85,2	76,8-90,9	4,1	
	50-59 años	80	1 200 193	95,1	83,9-98,6	3,3	
	≥60 años	110	2 021 789	98,8	91,6-99,8	1,2	
DIRIS/DIRESA							0,768
	Callao	235	957 804	81,4	75,4-86,2	3,3	
	Lima Centro	83	1 336 797	84,7	75,6-90,8	4,5	
	Lima Este	186	2 684 859	80,8	73,9-86,2	3,9	
	Lima Norte	149	2 316 697	84,2	77,7-89,1	3,4	
	Lima Sur	117	1 899 676	79,7	69,5-87,1	5.6	

CV: Coeficiente de variación, DIRIS: Direcciones de Redes Integradas de Salud, DIRESA: Dirección Regional de Salud.

a Estimación poblacional obtenida mediante los pesos muestrales.

b Prueba chi cuadrado corregida para diseño complejo.

La seroprevalencia de anticuerpos IgG contra el sarampión fue similar según sexo. En mujeres fue de 82,0% (IC95%: 78,1%-85,4%; CV: 2,3%), y en hombres de 82,1% (IC95%: 76,6%-86,5%; CV: 3,1%), no evidenciándose diferencias estadísticamente significativas entre ambos grupos (tabla 2).

La seroprevalencia de anticuerpos IgG contra el sarampión en la población evaluada mostró un patrón heterogéneo según grupo etario. Las menores seroprevalencias se observaron en los grupos de 20 a 29 años (55,6%; IC95%: 45,9%-64,7%; CV: 8,6%) y de 15 a 19 años (72,0%; IC95%: 58,1%-82,7%; CV: 8,7%). A partir de los 30 años se evidenció un incremento progresivo de la seroprevalencia, alcanzando 76,7% en el grupo de 30 a 39 años, 85,2% en el grupo de 40 a 49 años, 95,1% los de 50 a 59 años y 98,8% en las personas de 60 años a más. Todas las estimaciones presentaron coeficientes de variación menores al 10% (tabla 2, material suplementario).

La seroprevalencia de anticuerpos IgG contra el sarampión también fue similar entre las DIRIS y DIRESA evaluadas. Las estimaciones mayores se registraron en Lima Centro (84,7%; IC95%: 75,6%-90,8%; CV: 4,5%), y Lima Norte (84,2%; IC95%: 77,7%-89,1%; CV: 3,4%), seguidas por el Callao (81,4%; IC95%: 75,4%-86,2%; CV: 3,3%) y Lima Este (80,8%; IC95%: 73,9%-86,2%; CV: 3,9%). La menor seroprevalencia se observó en Lima Sur, con 79,7% (IC95%: 69,5%-87,1%; CV: 5,6%). En conjunto, las estimaciones oscilaron entre 79,7% y 84,7% sin diferencias significativas (tabla 2, material suplementario).

## DISCUSIÓN

Durante los últimos años, las coberturas de vacunación contra el sarampión han disminuido notablemente a nivel mundial, comprometiendo la inmunidad colectiva contra esta enfermedad y favoreciendo la reaparición de brotes en todos los continentes, incluyendo la región de las Américas ^16^.

En este contexto, la seroprevalencia general de anticuerpos IgG contra el sarampión observada en nuestro estudio fue inferior al umbral de inmunidad poblacional recomendado por la OMS (95%) para interrumpir la transmisión sostenida del virus y mantener el estado de eliminación de la enfermedad ^17^. Este hallazgo evidencia la persistencia de brechas de inmunidad en la población estudiada, ya que aproximadamente uno de cada cinco individuos no presentó anticuerpos IgG detectables contra el sarampión. Si bien la ausencia de IgG detectable no implica necesariamente susceptibilidad absoluta debido al papel de la inmunidad celular y de anticuerpos neutralizantes no evaluados en este estudio, los resultados sugieren la existencia de grupos poblacionales potencialmente vulnerables a la infección y a la propagación del virus en caso de exposición ^18^.

Los resultados obtenidos son comparables con los reportados recientemente en otros países. En México se estimó una seroprevalencia global de 78,3% ^19^, mientras que en Costa Rica la seropositividad alcanzó 74,8% ^20^. De manera similar, estudios realizados en Italia y España reportaron seroprevalencias de 91,2% y 92,2% respectivamente ^(^^21^^,^^22^. En conjunto, estos hallazgos evidencian que las brechas de inmunidad frente al sarampión persisten incluso en países que han alcanzado o mantenido la eliminación de la enfermedad, lo que pone de manifiesto la necesidad de fortalecer las estrategias de vacunación y monitoreo de la inmunidad poblacional.

Al analizar la seroprevalencia de anticuerpos IgG contra el sarampión según grupos de edad, se observó una marcada heterogeneidad. Los adolescentes y adultos menores de 40 años presentaron los niveles más bajos de seropositividad, siendo el grupo de 20 a 29 años el que registró la menor proporción de anticuerpos detectables. A partir de este grupo, la seroprevalencia aumentó progresivamente con la edad, superando el 95% en las personas de 50 años o más. Estos hallazgos son de gran relevancia para la salud pública, ya que los adultos jóvenes suelen presentar una mayor movilidad e interacción social debido a actividades laborales, académicas, migratorias y turísticas, lo que podría favorecer la introducción y propagación del virus. Asimismo, esta distribución es consistente con los últimos reportes de la sala situacional de sarampión del Centro Nacional de Epidemiología, Prevención y Control de Enfermedades, (CDC Perú), que muestran una mayor proporción de casos confirmados en adultos jóvenes ^6^. Un patrón similar ha sido descrito en los estudios de seroprevalencia realizados en Costa Rica, México e Italia ^19^.

Diversos factores podrían explicar la menor seropositividad observada en adolescentes y adultos jóvenes. Estas cohortes nacieron durante un período de transición epidemiológica caracterizado por una disminución progresiva de la circulación del virus del sarampión como consecuencia de la introducción y expansión de la vacunación ^20^. En consecuencia, es probable que hayan tenido una menor exposición al virus circulante y, por consiguiente, menos oportunidades de experimentar fenómenos de refuerzo inmunológico natural. En el Perú, la vacuna contra el sarampión fue incorporada al Programa Ampliado de Inmunizaciones (PAI) en la década de 1970 ^23^^,^^24^; como resultado, una proporción importante de estas cohortes habría desarrollado su inmunidad principalmente a través de la vacunación. A diferencia de la infección natural, la inmunidad inducida por la vacuna puede asociarse a una disminución progresiva de los niveles de anticuerpos detectables con el tiempo en ausencia de exposiciones posteriores al virus, y que un resultado negativo no indica necesariamente ausencia de protección ^25^. Adicionalmente, podrían haber contribuido otros factores, como esquemas de vacunación incompletos, considerando que la introducción sistemática de dos dosis de vacuna SRP se introdujo masivamente en el Perú recién en el año 2006 ^26^, así como fallas primarias de respuesta vacunal.

En contraste, la elevada seropositividad observada en los adultos de mayor edad podría explicarse por la inmunidad adquirida tras la infección natural, la cual se caracteriza por respuestas humorales más robustas y duraderas que pueden persistir durante toda la vida ^27^. Esto probablemente se debe a que dichas cohortes nacieron en periodos de alta circulación viral, previo a la introducción de la vacunación contra el sarampión, por lo que tuvieron una mayor probabilidad de exposición e infección durante la infancia ^28^. Además, como se mencionó anteriormente, la vacuna contra el sarampión recién estuvo disponible durante la década de 1970, por lo que gran parte de las personas actualmente mayores de 50 años crecieron en un contexto de circulación endémica del virus. Hallazgos similares han sido reportados en estudios realizados en Tailandia, México y Costa Rica, donde los adultos mayores presentaron las mayores proporciones de seropositividad en comparación con otros grupos etarios ^29^. Al analizar por sexo no se encontraron diferencias estadísticamente significativas en la seroprevalencia de participantes varones y mujeres, ni tampoco al evaluar por lugar de procedencia de los participantes.

Los resultados de este estudio evidencian brechas de inmunidad frente al sarampión en jóvenes y adultos de Lima Metropolitana y Callao. Aunque la ausencia de anticuerpos IgG detectables no implica necesariamente susceptibilidad absoluta, los hallazgos sugieren la existencia de cohortes con niveles de protección inferiores a los recomendados para prevenir la transmisión sostenida del virus. En el contexto actual de transmisión comunitaria en el Perú, esta situación podría incrementar el riesgo de ocurrencia y propagación de brotes.

Por lo cual, estos hallazgos resaltan la necesidad de priorizar las estrategias de vacunación en los grupos con mayores brechas de inmunidad, particularmente los adultos jóvenes, manteniendo al mismo tiempo altas coberturas en la población infantil. Asimismo, evidencian que la recuperación y sostenibilidad de la eliminación del sarampión requieren no solo coberturas de vacunación elevadas y homogéneas, sino también sistemas robustos de vigilancia epidemiológica y respuesta oportuna ante brotes.

Así, la reemergencia del sarampión revela que, para recuperar el estatus de eliminación de esta enfermedad a nivel regional, es primordial, más no suficiente, mantener coberturas de vacunación altas y homogéneas. Estas coberturas deben sostenerse en el tiempo y complementarse con una vigilancia epidemiológica y laboratorial robusta, sistemas de alerta-respuesta efectivos ante brotes y emergencias, la promoción de la eliminación mundial de la enfermedad y el mantenimiento de las campañas de seguimiento ^30^. Estas estrategias, adoptadas por la OPS en 1996, permitieron interrumpir la circulación endémica del virus en gran parte de las Américas y alcanzar el número más bajo de casos registrado hasta entonces en la región ^(^^31^.

En esa línea, los estudios de seroprevalencia son fundamentales ya que proporcionan información sobre la inmunidad poblacional, así como una estimación sobre la carga de enfermedad. Además, contribuyen en la evaluación de la eficacia de los programas de inmunización al actuar como indicadores indirectos de coberturas vacunales, facilitando la identificación de brechas de inmunidad en grupos específicos y orientando intervenciones de vacunación focalizadas en esta población ^(^^20^^,^^32^. En ese sentido, estos estudios deberían replicarse en otras regiones del Perú para obtener una estimación más precisa de la seroprevalencia a nivel nacional.

El presente estudio presenta algunas limitaciones que se describen a continuación. Primero, el análisis corresponde a una submuestra de participantes de la ENDES 2024 que aceptaron voluntariamente participar en la Vigilancia Laboratorial de Enfermedades No Transmisibles del INS. Aunque el diseño original de la ENDES fue probabilístico y los análisis incorporaron factores de expansión específicos ajustados por no respuesta y calibrados con proyecciones poblacionales según sexo y edad, no puede descartarse completamente un sesgo de selección o de no respuesta residual. Segundo, la captación de participantes se desarrolló entre mayo y setiembre de 2024 y no durante todo el periodo de recolección de la ENDES 2024. Si bien los conglomerados seleccionados estuvieron distribuidos geográficamente y a lo largo del periodo considerado de manera homogénea, no puede excluirse completamente la existencia de diferencias entre los participantes incluidos y aquellos entrevistados durante otros meses del año. Tercero, las estimaciones obtenidas son representativas de la población de 15 años o más de Lima Metropolitana y Callao considerada en conjunto y no necesariamente de cada DIRIS o de la DIRESA Callao de manera independiente. En consecuencia, las estimaciones desagregadas por estas jurisdicciones deben interpretarse con cautela como análisis de subgrupos. De igual manera, los resultados no deben extrapolarse directamente al resto del Perú debido a las diferencias regionales en estructura demográfica, cobertura de vacunación, condiciones socioeconómicas, movilidad poblacional y patrones históricos de circulación del sarampión. Cuarto, el diseño transversal del estudio refleja la inmunidad poblacional durante la segunda mitad del año 2024 y los primeros cuatro meses del 2025, lo que podría no reflejar la realidad actual. Quinto, la evaluación de la inmunidad se realizó mediante la detección de anticuerpos IgG por el método de ELISA, técnica ampliamente usada en estudios seroepidemiológicos, incluyendo COVID-19 ^33^, no habiéndose incluido anticuerpos neutralizantes ni inmunidad celular, por lo que los resultados podrían subestimar la protección inmunológica real de algunos individuos. Por último, no se contó con información concerniente al antecedente vacunal contra el sarampión de la población estudiada, por lo que no se pudo establecer con seguridad si la seropositividad observada obedeció a una respuesta por infección natural o por vacunación.

Sin embargo, una de las fortalezas principales de este estudio es que constituye la fuente de información más reciente de seroprevalencia contra el sarampión en el Perú, lo cual resulta de gran importancia en el contexto epidemiológico actual. Asimismo, incluye principalmente población joven y adulta, grupos poco estudiados en investigaciones de seroprevalencia de sarampión, tanto a nivel nacional como internacional. Finalmente, el análisis estratificado por grupos etarios permitió identificar brechas de inmunidad en cohortes específicas, generando evidencia útil para orientar y priorizar estrategias de vacunación focalizadas.

En conclusión, los resultados de este estudio indican que los adultos jóvenes constituyen un grupo importante de individuos susceptibles al presentar los niveles más bajos de inmunidad contra el sarampión, particularmente las personas de 20 a 29 años de edad. Estos hallazgos resaltan la necesidad de priorizar estrategias de prevención dirigidas a esta población, que incluyan intervenciones de vacunación focalizadas y el fortalecimiento de la vigilancia epidemiológica mediante la búsqueda activa de casos a nivel nacional, y campañas de información que promuevan la confianza en las vacunas. El cierre oportuno de estas brechas de inmunidad será fundamental para controlar el brote actual en nuestro país, detener la transmisión endémica del sarampión y preservar el estatus de eliminación sostenida alcanzado hasta el momento.
